# Supplementary material for: The T4bSS of Legionella features a two-step secretion pathway with an inner membrane intermediate for secretion of transmembrane effectors
Source: PLoS Pathog. 2024 Nov 15;20(11):e1012118. doi: 10.1371/journal.ppat.1012118 (PMC11602083; doi:10.1371/journal.ppat.1012118)
Supplement: S1 Fig — The relative abundance of the indicated T4bSS components in 12 fractions of a membrane-fractionating sucrose gradient as analyzed by mass spectrometry. The major coupling complex components as well as DotO follow the distribution of known inner membrane proteins (see Fig 1A). The minor coupling complex components IcmS, IcmW and LvgA also follow a distribution of known inner membrane proteins but show an additional tendency to co-fractionate with outer membranes. DotB follows the distribution of soluble proteins and the core complex components follow the distribution of outer membrane proteins. The equilibration of the core complex in the outer membrane fractions may result from the high density of vesicles that contain the large core complex. Also, a fully assembled coupling complex including the minor components may be of sufficiently high density to co-fractionate with outer membranes. Alternatively, it is conceivable that fully assembled coupling complexes interact with the core complex and are therefore found in the fractions of high density. (PDF) [file ppat.1012118.s007.pdf]

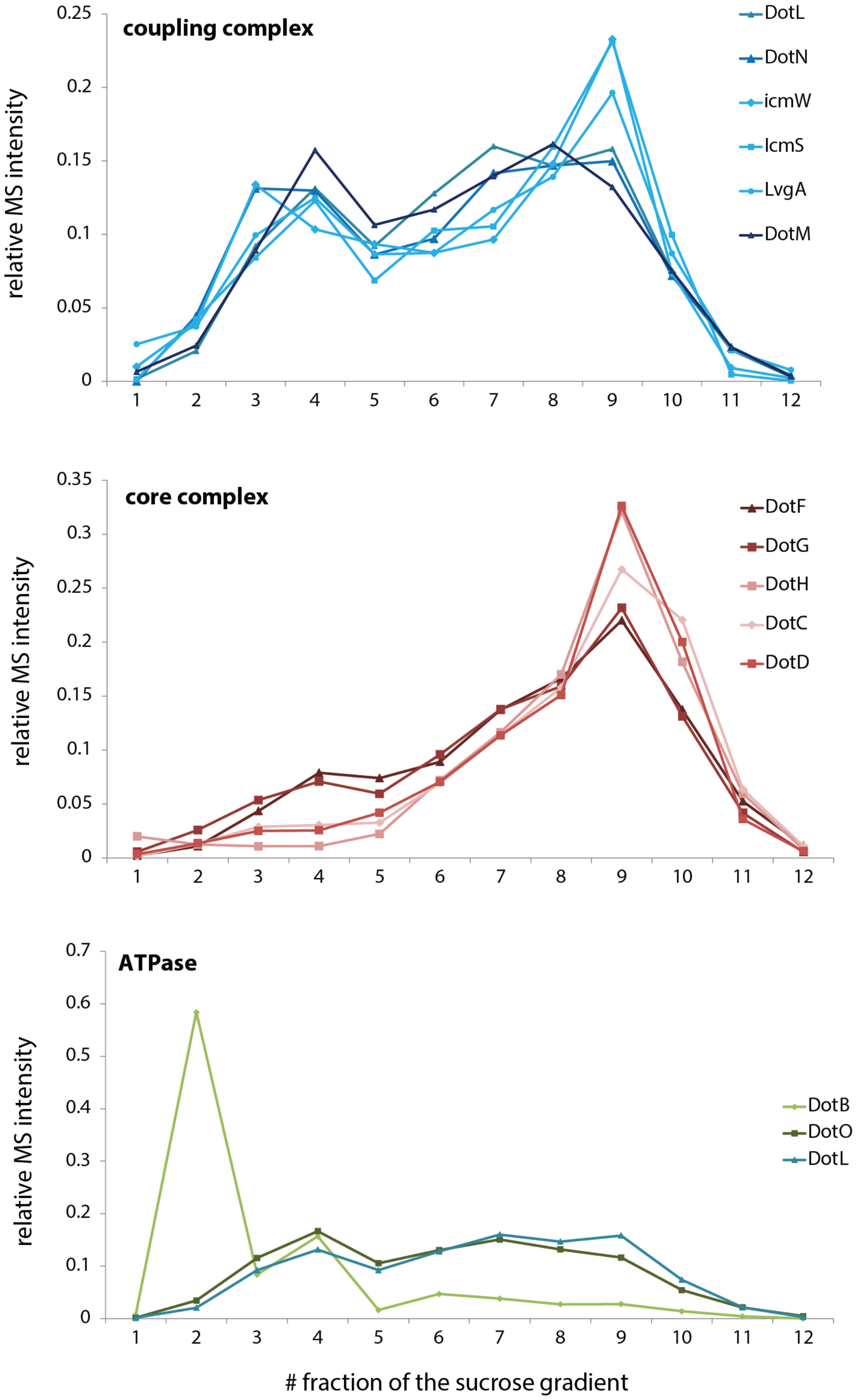

S1 Fig. Distribution of T4bSS components across the membrane-fractionating sucrose gradient

The relative abundance of the indicated T4bSS components in 12 fractions of a membrane-fractionating sucrose gradient as analyzed by mass spectrometry. The major coupling complex components as well as DotO follow the distribution of known inner membrane proteins (see Fig 1A). The minor coupling complex components IcmS, IcmW and LvgA also follow a distribution of known inner membrane proteins but show an additional tendency to co-fractionate with outer membranes. DotB follows the distribution of soluble proteins and the

core complex components follow the distribution of outer membrane proteins. The equilibration of the core complex in the outer membrane fractions may result from the high density of vesicles that contain the large core complex. Also a fully assembled coupling complex including the minor components may be of sufficiently high density to co-fractionate with outer membranes. Alternatively it is conceivable that fully assembled coupling complexes interact with the core complex and are therefore found in the fractions of high density.
