# Supplementary material for: The T4bSS of Legionella features a two-step secretion pathway with an inner membrane intermediate for secretion of transmembrane effectors
Source: PLoS Pathog. 2024 Nov 15;20(11):e1012118. doi: 10.1371/journal.ppat.1012118 (PMC11602083; doi:10.1371/journal.ppat.1012118)
Supplement: S2 Fig — The accumulation levels of the indicated plasmid-expressed and epitope-tagged effector proteins in the L. pneumophila wild type were analyzed by SDS-PAGE, Western blotting and immunodetection. (PDF) [file ppat.1012118.s008.pdf]

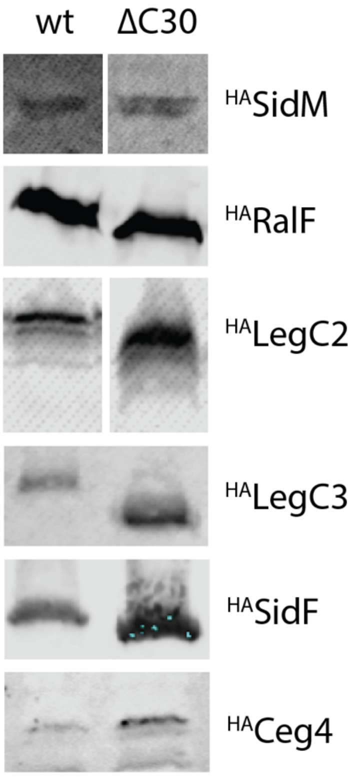

S2 Fig. Accumulation levels of effector proteins with and without the 30 C-terminal amino acids

The accumulation levels of the indicated plasmid-expressed and epitope-tagged effector proteins in the *L. pneumophila* wild type were analyzed by SDS-PAGE, Western blotting and immunodetection.
